# Supplementary material for: Differential Accumulation of Metabolites and Transcripts Related to Flavonoid, Styrylpyrone, and Galactolipid Biosynthesis in Equisetum Species and Tissue Types
Source: Metabolites. 2022 Apr 29;12(5):403. doi: 10.3390/metabo12050403 (PMC9146389; doi:10.3390/metabo12050403)
Supplement: Supplementary file 1 [file metabolites-12-00403-s001.zip › ParrishEtAl_EquisetumMultiOmics_SupplFigS1.pdf]

Supplementary Figure S1

Parrish et al. (2022), Metabolites

Translated peptide sequence alignment for selected monogalactosyldiacylglycerol synthases (MGDs) and digalactosyldiacylglycerol synthases (DGDs). Species abbreviations: A.t., Arabidopsis thaliana; E.a., Equisetum arvense; E.h., Equisetum hyemale; E.t., Equisetum telmateia.

```
A.t.MGD2      ------------------------------------------------------------   0
A.t.MGD1      ------------------------------------------------------------   0
E.t.MGD       ------------------------------------------------------------   0
E.h.MGD       ------------------------------------------------------------   0
A.t.DGD1      MVKETLIPPSSTSMTTGTSSSSSLSMTLSSTNALSFLSKGWREVWDSADADLQLMRDRAN   60
A.t.DGD2      ------------------------------------------------------------   0
E.h.DGD       ------MAP--------RGTDSHTTIPASAEKALSLISKSWFDVRKSANEDVKRMKDRAI   46
E.a.DGD       ------MAP--------WATDSHTTIPASAEKALSLISKSWFDVRKSANEDVKRMKDCAI   46


A.t.MGD2      ------------------------------------------------------------   0
A.t.MGD1      ------------------------------------------------------------   0
E.t.MGD       ------------------------------------------------------------   0
E.h.MGD       ------------------------------------------------------------   0
A.t.DGD1      SVKNLASTFDREIENFLNNSARSAFP------------VGSPSASS--FSNEIGIMKKLQ   106
A.t.DGD2      ------------------------------------------------------------   0
E.h.DGD       SFSDLANSFDRELENFRKSSLKIANNSSIAERFLQKFQNGETLLPSLSVSVPEEVNNKLR   106
E.a.DGD       SFSDLANSFDRELENFRKSSLKIANNASIAERFLQKFQNGGTLLPSLNVSVPEEVNNKLK   106


A.t.MGD2      ------------------------------------------------------------   0
A.t.MGD1      ----------------------MQ--NPSTV---TQESA-----AP----------VF-D   17
E.t.MGD       -------RVCGRGER-----ERMQ--RPSDA---LPQHS-----IPFTSAGDLEAPLIID   38
E.h.MGD       ------------------------------------------------------------   0
A.t.DGD1      PKISEFRRVYSAPEISRK---VMERWGPARAKLGMDLSAIKKAIVSEMELDE---RQGVL   160
A.t.DGD2      ------------------------------------------------------------   0
E.h.DGD       ISFSGFSRSYSNPEFVKRRADFLHKWAPSKK---LDVSLIKKVLSPRIDEEDFYIKTGSE   163
E.a.DGD       ISFSGFSRSYSNPDFVKRQADFIHKWAPSKK---LDVSLIKKVLSPRIDDEDFYIKTGAE   163


A.t.MGD2      ------------------------------------------------------------   0
A.t.MGD1      FFPRLR-GLTSRNRSPCSNSDGYA---------------------------LSSSNALYF   49
E.t.MGD       HSCFLGGGGEASAGRPCVGDYSGR---------------------------PRSVVVCCL   71
E.h.MGD       -----------------VGPYGGR---------------------------PRGVVVHCL   16
A.t.DGD1      EMSRL-------RRRRNSDRVRFTEFF--------AEAERDGEAYFGDWEPIRSLKSRF-   204
A.t.DGD2      ------------------------------------------------------------   0
E.h.DGD       VISSGKGGHHGNHRNNHTQRLVVKSWDKDNKNTKNEG-DGQGSLTNQEWELLRRFKESWW   222
E.a.DGD       IISSGKGGHHGNCQNNCSQRVVVKSWANNNKHTKNEGDDGRGNLTNQEWELLRRFKESWW   223


A.t.MGD2      ----------------------------MATTVMALA-----------------------   9
A.t.MGD1      N-----------------GF-RTLPSRRMGKTLASL---SFNTKSSA--GSSL-------   79
E.t.MGD       RR--------------CRGNAKPKPRPR-AKLILAMSASPWD-------RPPLSP-----   104
E.h.MGD       RR--------------CVGNAKPKSFSR-AKAILAMSASPQDGFDQFVNRSLHSP-----   56
A.t.DGD1      KEFE-----KRSSLEILSGFKNSEFVEKLKTSF-KS---IYKETDEAKDVPPLDVPELLA   255
A.t.DGD2      ------------------------------------------------------------   0
E.h.DGD       KDLETTAAVNRSPSDFFDNM-RTEVFENIKKNL-RL---TVPRFEDGKDVAPLDVSELLA   277
E.a.DGD       KDLETTVAVNKSPSDFFDNITKTEIFENLKKNL-RL---TVPRFEDGKDVAPLDVSELLA   279


A.t.MGD2      -----------------EKVLERVYGTSKSAVSVTSGDGEKTHRHTHHHIHRIKS---YD   49
A.t.MGD1      --RRFISDFNSFIRFHCDKVVPESF------ASVGGVGLSSDEN----GIREN-G----T   122
E.t.MGD       --PRGGAAFLASLGLRKDELL----------SSLANGDAAKDCN----DAEAA-A---PP   144
E.h.MGD       --LRGGTAFLASLGLRQDEF-----------SSLANDDIVKDCN----DGEAA-E---PP   95
A.t.DGD1      CLVRQSEPFLDQIGVRKDTCDRIVE-------SLCKCKSQQ-----L---WRLPSAQASD   300
A.t.DGD2      ------------------------------------------------------------   0
E.h.DGD       ILIRQSESWLDHLGLKKDVVERVCD-------MLVKGRHKNKKE-QSHSIFNGNKEALAT   329
E.a.DGD       ILIRQSEPWLDYLGLKKDVVERVCD-------MLVKGRHKNKKE-QSHSIFNGNKEVLAT   331
```

Supplementary Figure S1 (continued)

Parrish et al. (2022), Metabolites

```
A.t.MGD2      DIDEDESSLE----LIQI------GAERTKNVLILMSDTGGGHRA---------------   84
A.t.MGD1      GGVLGEEGLP----LNGV------EADRPKKVLILMSDTGGGHRA---------------   157
E.t.MGD       ENMTVDGNAD----GAGV------VPRRRKTVLILMSDTGGGHRA---------------   179
E.h.MGD       ENVAVDENTN----EAGV------VPRRRKTVLILMSDTGGGHRA---------------   130
A.t.DGD1      LIENDNHGVDLDMRIASVLQSTGHHYDGGFWTDFVKPETPENKRHVAIVTTASLPWMTGT   360
A.t.DGD2      -------------------------------------MTNQQEQHIAIFTTASIPWLTGT   23
E.h.DGD       VKDTGKSMEELDFRLASVMQSTGYKYNGRTWGGQIKKEVESGMRNIAIFTTASLPWMTGT   389
E.a.DGD       MKDTGKNMEELDFRLASVMQSTGYNYNGRTRGGQSMKDVETGMRNIAIFTTASLPWMTGT   391
                                                    .    :

A.t.MGD2      -SAEAIRDAFKIEFGDKYRVIVKDVWKEYT----GWPL---------------NDMERSY   124
A.t.MGD1      -SAEAIRAAFNQEFGDEYQVFITDLWTDHT----PWPF---------------NQLPRSY   197
E.t.MGD       -SAEAIKATFQLEFGDEYEVLITDLWKEHT----PWPF---------------NQLPRSY   219
E.h.MGD       -SAEAIKATFQLEFGDEYEVVITDLWKEYT----PWPF---------------NQLPRSY   170
A.t.DGD1      AVNPLFRAAYLAKAAKQ-SVTLVVPWLCESDQELVYPNNLTFSSPEEQESYIRKWLEERI   419
A.t.DGD2      AVNPLFRAAYLANDGER-RVTLVIPWLTLKHQKLVYPNSITFSSPSEQEAYVRQWLEERV   82
E.h.DGD       AVNPLLRAAYLAKNGKQ-NVTLLVPWLCKKEQKLVYPNNMNFDSPEEQEAYVRNWLESRL   448
E.a.DGD       AVNPLLRAAYLAKNGKQ-NVTLLVPWLCKKDQKFVYPNNMTFDSPEEQEGYVRNWLESRL   450
                   :: ::  : ...  * :   *   .     :*                : :

A.t.MGD2      KFMVKHVQLWKVAFHSTSPKWIHSCYLAAIAAYYAKEVEAGLMEYKPEIIISVHPLMQHI   184
A.t.MGD1      NFLVKHGTLWKMTYYGTSPRIVHQSNFAATSTFIAREIAQGLMKYQPDIIISVHPLMQHV   257
E.t.MGD       GFLVKHSTLWKATYHVTAPRIVHRTHFAVTSTFIARKVMQGLLKHKPDVIVSVHPLMQHI   279
E.h.MGD       GFLVRHSTLWKATYHVTAPRIVHRTHFAVTSTFIARKVMQGLLKHKPDVIVSVHPLMQHI   230
A.t.DGD1      GFK----ADFKISFYPGKFSKERRSIF------PAGDTSQFISSKDADIAILEEPEHLNW   469
A.t.DGD2      SFR----LAFEIRFYPGKFAIDKRSIL------PVGDISDAIPDEEADIAVLEEPEHLTW   132
E.h.DGD       GFK----SDFKIAFYPGKFSKQKRSIL------ACGDISQFISDEEADIAVLEEPEHLTW   498
E.a.DGD       GFK----SDFKIAFYPGKFSKQKRSIL------ACGDISQFVSDQEADIAVLEEPEHLTW   500
               *       ::  ::       :   :         .    : . . :: :  .*

A.t.MGD2      PLWVLKWQELQKRVLF---VT----VIT----DLNTCHPTWFHPGVNRCYCPSQE-----   228
A.t.MGD1      PLRVLRSKGLLKKIVF---TT----VIT----DLSTCHPTWFHKLVTRCYCPSTE-----   301
E.t.MGD       PLRILKARGLLKKIPF---TT----VIT----DLSTCHPTWFHRLVTRCFCPTKE-----   323
E.h.MGD       PLRILKARGLLKKIPF---TT----VIT----DLSTCHPTWFHRLVTRCFCPTKE-----   274
A.t.DGD1      YYHGKRWTDKFNHVVGIVHTNYLEYIKREKNGALQAFFVNHVNNWVTRAYCDKVLRLSAA   529
A.t.DGD2      FHHGQKWKTKFNYVIGIVHTNYLEYVKREKQGRVKAFFLKYLNSWVVGIYCHKVIRLSAA   192
E.h.DGD       YFHGKRWTDKFNYVVGVVHTNYLEYVKREKNGALQAFLLKHVNNWVTRIYCHKVLRLSAA   558
E.a.DGD       YFHGKRWTDKFKYVVGVVHTNYLEYVKREKNGALQAFLLKHVNNWVTRIYCDKVLRLSAA   560
                   :     : :     ..    :       :.:   . .:  *   :* .

A.t.MGD2      --------------VAKRALFDGLDESQVRVFGLP---------VRPSFARAVLVKDDLR   265
A.t.MGD1      --------------VAKRAQKAGLETSQIKVYGLP---------VRPSFVKPVRPKVELR   338
E.t.MGD       --------------VAEKALKAGLKTTQIRVYGLP---------IRPSFCKDIPPKVDLR   360
E.h.MGD       --------------VAEKALKAGLKTTQIRVYGLP---------IRPSFCKDIPPKVELR   311
A.t.DGD1      TQDLPKSVVCNVHGVNPKFLMIGEKIAEERSRGEQAFSKGAYFLGKMVWAKGYRELIDLM   589
A.t.DGD2      TQEYPKSIVCNVHGVNPKFLEIGLRKLEQQKLQEQPFTKGAYYIGKMVWSKGYKELLKLL   252
E.h.DGD       TQELPRSIVCNVHGVNPKFLEIGEKMAQDGKGSQDTFSKGAYFLGKMVWGKGYRELVDLL   618
E.a.DGD       TQELPRSIVCNVHGVNPKFLEIGEKMVQEGEGSQDTFSKGAYFLGKMVWGKGYRELVDLL   620
                            *  :    *    :                 :  : :      .*

A.t.MGD2      KELEMDQDLRAVLLMGGGEGMGPVKETAKALEEFLYDKENRKPIGQMVVICGRNKKLASA   325
A.t.MGD1      RELGMDENLPAVLLMGGGEGMGPIEATARALADALYDKNLGEAVGQVLIICGRNKKLQSK   398
E.t.MGD       RSLGMNEELPAVLLMGGGEGMGPVESTAKALGESLFCQQSGEAIGQLVVICGRNTKLVKN   420
E.h.MGD       RALGMNEELPAVLLMGGGEGMGPVESTAKALGESLFCQQSGEAIGQLVVICGRNMKLVKN   371
A.t.DGD1      AKHKSELGSFNLDVYGNGEDAVEVQRAAKKHDLNLNFLKGRDHADDAL------------   637
A.t.DGD2      EKHQKELAELEVDLYGDGEDSEEIKEAARKLDLTVNVYPGRDHADSLF------------   300
E.h.DGD       AKHKSDLGSLKIDVFGTGEDSEEVKATAKKLGLDLNFHQGRDHADDCL------------   666
E.a.DGD       AKHKSDLGSLKIDVFGTGEDSEEVKATAKKLGLDLNFNQGRDHADDSL------------   668
                   :     : : * **.   :: :*:     :      .  .. .
```

Supplementary Figure S1 (continued)

Parrish et al. (2022), Metabolites

```
A.t.MGD2      LEAIDWKIPVKVRGFETQMEKWMGACDCIITKAGPGTIAESLIRSLPIILNDYIPGQEKG  385
A.t.MGD1      LSSLDWKIPVQVKGFITKMEECMGACDCIITKAGPGTIAEAMIRGLPIILNGYIAGQEAG  458
E.t.MGD       LQSINWSMPVQIRGFEKNIEEWMGACDCLITKAGPGTIAEAMIRGLPLILSDYIAGQEVG  480
E.h.MGD       LQSINWSIPVQIRGFEKNIEEWMGACDCLITKAGPGTIAEAMIRGLPLILSDYIAGQEVG  431
A.t.DGD1      ------------HKYKVFINPSISDVLC-------TATAEALAMGKFVVCADHPSNEFFR  678
A.t.DGD2      ------------HNYKVFLNPSTTDVVC-------TTTAEALAMGKIVVCANHISNKFFK  341
E.h.DGD       ------------HGYKVFINPSISDVVC-------TTTAEALAMGKIVVCADHPSNEFFR  707
E.a.DGD       ------------HGYKVFINPSISDVVC-------TTTAEALAMGKIVVCADHPSNDFFR  709
                          : :   ::       *        : **::  .  ::  .:  ..


A.t.MGD2      NVPYVVENGAGVFTRSPKETARIVGEWFSTKTDELEQTSDNARKLAQPEAVFDIVKDIDE  445
A.t.MGD1      NVPYVVENGCGKFSKSPKEISKIVADWFGPASKELEIMSQNALRLAKPEAVFKIVHDMHE  518
E.t.MGD       NVPFVVDNGAGKFLKEPKKIAQTVADWFGPKREELHTMSRNALKLARPDAVFKIVHDLDK  540
E.h.MGD       NVPFVVDNGAGKFLKEPKKIAQTVADWFGPKREELHIMSRNALKLARPDAVFKIVHDLDR  491
A.t.DGD1      SFPN-----CLTYKTS-EDFVSKVQEAMTKEPLPLTPEQMYNLSWEAATQRFMEYSDLDK  732
A.t.DGD2      QFPN-----CRTYDDG-QGFVRATLKALGEQPSQLTEQQRHELSWEAATQRFIKVSDLNR  395
E.h.DGD       SFPN-----CLTYKDS-REFVEKVKEAMAIDPAPLTLEQRHLLSWEAATERFIEYAELDK  761
E.a.DGD       SFPN-----CLTYKDS-REFVEKVKEAMDIDPVPLTPEQRHLLSWEAATERFIEYAEIDK  763
              ..*      .  :    .     . . :      *   .            *    ::..

A.t.MGD2      LSEQRGPL-------------------ASVSYNLTSSF--ASLV----------------  468
A.t.MGD1      LVRKKNSL-------------------PQLSCTA--------------------------  533
E.t.MGD       LARRKVFL-------------------EENRTLVPT------------------------  557
E.h.MGD       LARRKVFL-------------------EENRTLVAT------------------------  508
A.t.DGD1      ILNNGEGG----------RKMRKSRSVPSFNEVVDGGLAFSHYVLTGNDFLRLCTGATPR  782
A.t.DGD2      LSRADSN--LSKRSVFASSSISV-------GKNLEDMSAYIHFLASGFEASRTAFGAIPG  446
E.h.DGD       PFIKQGGESVTPEKVVQKKAMALSMAMPNPFELVDNSLAAAHYCLTGIEAARFISGALPG  821
E.a.DGD       PFIKQGGESVTPENVVQKKAMALSMAIPNPSELVDNSLAAAHYCLTGIEAARLISGALPG  823


A.t.MGD2      ----------------------------      468
A.t.MGD1      ----------------------------      533
E.t.MGD       ----------------------------      557
E.h.MGD       ----------------------------      508
A.t.DGD1      TKDYDNQHCKDLNLVP--PHVHKPIFGW      808
A.t.DGD2      SLQPDEELCRDLGLSLNTPSPNTRKQD-      473
E.h.DGD       SINYDSQHSKDLHLQP--PSVEKPVYGW      847
E.a.DGD       SSNYDSQHSKDLHLQP--PSVERPVYGW      849
```

Supplementary Figure S1 (continued)

Parrish et al. (2022), Metabolites

Translated peptide sequence alignment for a styrylpyrone synthase (SPS), a p-coumaroyltriacetic acid synthase (CTAS), selected chalcone synthases (CHSs), and a contig of transcriptome data for Equisetum telmateia (DN52675). Species abbreviations: C.p., Cyclosorus parasiticus; E.a., Equisetum arvense; E.h., Equisetum hyemale; E.t., Equisetum telmateia; P.M., Piper methysticum.

```
P.m.SPS         ------MSKTVEDRAAQRAKGPATVLAIGTATPANVVYQTDYPDYYFRVTKSEHMTKLKN         54
E.h.CTAS        --------------------------------PLTAYEQANYPDFYFGITNSNHMTDLKE         28
E.t.DN52675     ------------------------------------------------------------         0
C.p.CHS         MPVPNGATFPPCARKMERADGPATVLAIGTANPPNVFDQSTYPDFYFNITNSNHMTDLKT         60
E.a.CHS         MTVLEESADASSRRLAQRANGPATVLAIGTANPANVFEQSSYPDFYFDITNSQHMTELKL         60


P.m.SPS         KFQRMCDRSTIKKRYMVLTEELLEKNLSLCTYMEPSLDARQDILVPEVPKLGKEAADEAI         114
E.h.CTAS        KFSRMCEKSGIKKRYLHLTEEILKANPSMCGYWEKSLDVRQDIVVVEVPMLARQASIKAI         88
E.t.DN52675     -------------------------------------------VVVEVPKLAKEASLKVI         17
C.p.CHS         KFQRMCDKSGITKRYMYLNEEILKANPNMCAYWEKSLDVRQDMVVVEVPKLGKEAATKAI         120
E.a.CHS         KFSRMCQKSGIKKRYMHLNSEILKANPSLCAYWEKSLDVRQDIAVVEVPKLGKEASLKAI         120
                                                            * *** *.::*: :.*

P.m.SPS         AEWGRPKSEITHLIFCTTCGVDMPGADYQLTKLLGLRSSVRRTMLYQQGCFGGGTVLRLA         174
E.h.CTAS        KEWGQPKSKITHLVFCTTNGLDMPGADWKLTKLLGLHPNVKRLMIYQQGCFAGGTVMRIA         148
E.t.DN52675     KEWGQPKSKITHLIFCSTSGVDIPGADWQLTKILGLCPSVKRLMMYQQGCFAGGTVLRIA         77
C.p.CHS         KEWGQPKSKITHVVFCTTSGVDMPGADWALTKLLGLRPSVKRLMMYQQGCFAGGTVMRVA         180
E.a.CHS         KEWGQPKSKITHLVFCTTSGVDMPGADWALTKLLGLRPSVKRLMMYQQGCFAGGTVLRVA         180
                 ***:***:***::**:* *:*:****: ***:***  .*:* *:******.****:*:*

P.m.SPS         KDLAENNAGARVLVVCSEITTAVNFRGPSDTHLDLLVGLALFGDGAAAVIVGADPDPTLE         234
E.h.CTAS        KDLAENNKDARVLVVCSEIN-LSTFRGPSDIHLDSLVGQALIGDGASAMIIGSDPIPKVE         207
E.t.DN52675     KDLAENNKDARVLVVCSEIT-VVTFRGPSETNFANLVGQALFGDGVSAVIIGSDPIPQVE         136
C.p.CHS         KDLAENNKGARVLVVCSELT-AVTFRGPSETHLDSLVGQALFGDGASAIIVGADPIPEVE         239
E.a.CHS         KDVAENNKGARVLVVCSEIT-CVTFRGPSETHLDSLVGQALFGDGAAAVILGSDPLPE-E         238
                **:**** .*********:.   .*****: ::  *** **:***.:*:*:*:** *  *

P.m.SPS         RPLFQIVSGAQTILPDSEGAINGHLREVGLTIRLLKDVPGLVSMNIEKCLMEAFAPM---         291
E.h.CTAS        TPWFELHWSGSTILPESNGAIDGHLREVGLIFHLSKDVPRIISKNIGVLLTDAFEKAFL-         266
E.t.DN52675     TPWFEIHWSGSTILPDSDGAIDGHLREVGLTFHLREDVPRIISKNIGVVLNDAFDKVFP-         195
C.p.CHS         RPWFEIHYVASNILPDSDGAIDGHLREVGLTFHLMKDVPGIISKNIGTVLKDAFEKVFG-         298
E.a.CHS         NPCFELHWSGSNILPDSDGAIDGHLREVGLTFHLMKDVPGIISKNIGKVLNDAFRSAFDE         298
                 * *::   ...***:*:***:******** ::* :*** ::* **   * :**

P.m.SPS         ------GIHDWNSIFWIAHPGGPTILDQVEAKLGLKEEKLKSTRAVLREYGNMSSACVLF         345
E.h.CTAS        --GGKEAPPSYNDVFWIAHPGGPAILDQIEAKLQLKMEKMHASRSILSEYGNMSSASVIF         324
E.t.DN52675     --SGEGAPLSYNDVFWIAHPGGPAILDQIEEKLKLIPKKMQASRAILYEYGNMSSASVFF         253
C.p.CHS         --NEEGEVPSYNDVFWIAHPGGPAILDQVEQKLQLKTEKMAASRQVLSDYGNMSSACVLF         356
E.a.CHS         SGNAEDRPASVNDIFWIAHPGGPAILDQVEEKMKLAPEKMRATRDVLSEYGNMSSACVLF         358
                         . *.:*********:****:* *: *  :*: ::* :* :*******.*:*

P.m.SPS         ILDEVRKRSMEEGKTTTGEGFDWGVLFGFGPGFTVETVVLHSMPIPKADEGR       397
E.h.CTAS        IMDYMRQQAVEKKLATTGEGLDWGVLLGFGPGLTVETILLKSVNLCN-----       371
E.t.DN52675     IMDYLRKQSLENKLPTTGDGFDWGLLLGFGPGLTIETIVLKSVPINN-----       300
C.p.CHS         IMDHLRKKSVEQKLATSGEGYEWGLLLGFGPGLTCETVVLRSVPLATE----       404
E.a.CHS         IMDHMRRMSAQNKLQTTGEGLDWGVLLGFGPGLTVETVLLKSIRLAC-----       405
                *:* :*: : ::   *:*:* :**:*:*****:* **::*:*: :
```

Supplementary Figure S1 (continued)

Parrish et al. (2022), Metabolites

Translated peptide sequence alignment for phenylalanine ammoina lyases (PALs). Species abbreviations: E.a., Equisetum arvense; P.a., Pteridium aquilinum; P.e., Pellia epiphylla; 56P.n., Psilotum nudum.

```
P.e.PAL        ----------------------RLHGKNLLFVD--GPVAKPPHWAKAAEALQTSHYEEVR   36
E.a.PAL        MAAEFQNQKKLAELHRPPTLPLPVQGKNPLFVDSKEPVAKPPHWQKAAEALQSSHYEEIR   60
P.a.PAL        ---------------------------------------------------GGSFLDDIK   9
P.n.PAL        MTADLVL-PVLGIGSKPLGLAHTVCGGRLLP---HKSEGDPLKWVESAQESECSHLDEVR
                                                                    *. ::::

P.e.PAL        RMIRQFYESEKVILRGSSLTVGQVTAVTQRASVIVQLDEAEAKSRVDESSNWVLNRTLTG   96
E.a.PAL        RMIRQFLETEKVVLQGSSLTVGQVTAVTQRSEVAVELDEATAKARVDESSNWVLNNILKG   120
P.a.PAL        QMISTFTAAEELKIEGKTLTVAEVAAFARSKEMVLCLDEAAAKQRVDESALWVQNKIMKG   69
P.n.PAL        RMVKEFSDGNVVSLQGLNLTVAQVAAVARRPDVKVELDSAVARRRVDESSEWVVNHIMKG   116
               :*:  *   : : :.* .***.:*:*.::  .: : **.* *: *****: ** *. :.*

P.e.PAL        TDTYGVTTGFGATSHRRTNQVVDLQRELIRFLNAGVIGR-DSDGCSLPLKYARAAMLVRT   155
E.a.PAL        TDTYGVTTGFGATSHRRTTQAHDLQRELIRFLNAGVITAGKGANCTLPLPYAKAAMLVRT   180
P.a.PAL        CDVYGVTTGYGATSHRRTNQGIDLQRELIRFLNAGIFG--KSQANALPLDAARAAVLVRT   127
P.n.PAL        TDTYGVTTGFGATSHRRTNQGIELQKELIRFLNAGILTDGKEECNSLPTPAVRAAILVRT   176
                *.******:********.*  :**:*********::   .    :**   .:**:****

P.e.PAL        NTLMQGYSGIRWDILDAMRKLMCANVIPKLPLRGTITASGDLVPLSYIAGLLTGRSNSKA   215
E.a.PAL        NTLMQGYSGIRWAILNGFEKLMNGNIIPKMPLRGTITASGDLVPLSYIAGLITARPNSRA   240
P.a.PAL        NTLMQGYSGIRWEILQTIEKLLNANITPLLPLRGTITASGDLVPLSYLAGVLTGRPNSKA   187
P.n.PAL        NTLMQGFSGIRWAILEAMEKLLNSQITPRLPLRGTITASGDLVPLSYIAGLLTARPNSRA   236
               ******:***** **: :.**: .:: * :*****************:**::*.* **:*

P.e.PAL        ITPEGKEVSAAEALKIAGIDGPFELQPKEGLALVNGTSVGAAVAANVCFDANVLALLSEV   275
E.a.PAL        ITPSGEEIPAAEALKLVGIEEPFVLQPKEGLAIVNGTSVGAAVAANVCFDANVLVLLAEV   300
P.a.PAL        VTAEGKVVSGAEALKMVGVEKPFELQPKEGLAIVNGTAVGAGLASMVCFDAHILALLSVV   247
P.n.PAL        VTAEGKEVTGEEALKLAGIQEPFDLQPKEGLALVNGTAVGAGLAAVVCYDANIFALLAEV   296
               :* .*: : . ****:.*:: ** ********:****:***.:*: **:**:::.**: *

P.e.PAL        LSAFFCEVMQGKPEFTDPLTHQLKHHPGQIEAAAVMEFLLEGSSYMKAAAKLHETDPLSK   335
E.a.PAL        ISALFCEVMQGKPEFTDPLTHQLKHHPGQIEAAAVMEYLLEGSSYMQAAAKLHETDPLSK   360
P.a.PAL        TSAMFCEVMQGKPEFTDPLTHRLKHHPGQIEAAAAMEYLLEGSALVKAASKLHGVDALKK   307
P.n.PAL        LSALFCEVMQGKPEFTDHLTHRLKHHPGQIEAAAIMEWLLDGSSYMKAAAKLHETDPLKK   356
                **:************* ***:************ **:**:**: ::**:*** .* *.*

P.e.PAL        PKQDRYALRTSPQWLGPQIEVIRSATHSIEREINSVNDNPLIDVSRDMALHGGNFQGTPI   395
E.a.PAL        PKQDRYALRTSPQWLGPQAEVIRAATHCIEREINSVNDNPLIDVSRDMALHGGNFQGTPI   420
P.a.PAL        PKQDRYALRTSPQWLGPQIEVIRFATQLIQREINSVNDNPIIDVSRDLALHGGNFQGTPI   367
P.n.PAL        PKQDRYALRTSPQWLGPQIEVIRLATHAIQREINSVNDNPLIDVARDKALHGGNFQGTPI   416
               ****************** **** **: *:**********:***:** ************

P.e.PAL        GVSMDNMRIALAAIGKLMFAQFSELVCDYYNSGLPSNLSGGPNPSLDYGFKGAEIAMAAY   455
E.a.PAL        GVSMDNMRIALAAIGKLMFAQFSELVCDHYNSGLPSNLSGGPNPSLDYGFKGAEIAMAAY   480
P.a.PAL        GTAMDNIRLALAAIGKLMFAQFSELVNDFYNNGLPSNLSAGPNPSLDYGLKGGEIAMASY   427
P.n.PAL        GVSMDNMRLAIAAIGKLMFAQFSELVNDLYNNGLPSNLSGSPNPSLDYGFKGAEIALASY   476
               *.:***:*:*:*************** * **.*******..********:**.***:*:*

P.e.PAL        CSELQYLANPVTTHVQSAEQHNQDVNSLGLIAARKTAEAVEILKLMSSTYMVALCQAVDL   515
E.a.PAL        CSELQYLANPVTTHVQSAEQHNQDVNSLGLISARKTAEAIEILKLMSASYLVALCQAIDL   540
P.a.PAL        TSELEYLANPVTNHVQSAEQHNQDVNSLGLISARKTAEAVEVLKLMCATYLVGICQAIDL   487
P.n.PAL        TSEMQYLANPVTTHSQSAEQHNQDVNSLGLISARKTKEAIDILKLMISTYIVALCQAVDL   536
                **::*******.* ****************:**** **:::**** ::*:*.:***:**

P.e.PAL        RHLEENMQAVVKRLVESATKSTLYTENG-SLFPTRFSEKDLLQVVDHQPVFSYIDNASNP   574
E.a.PAL        RHLEENMQAIVKHVVKKVIKKSLYNVEGESLLPWAGAEKELLSIIDHQPVFSYIDNASNP   600
P.a.PAL        RHLEENLQAAVKQVVSQAAKKTLTTGGNGVLLAARFCEKDLLQVVDNEHVFTYADDPVSP   547
P.n.PAL        RHLEENMKAAIKHMVCQVARRTLYFDHNGLLLPSRFCEKELLHVVEHEPIFLYIDNASSD   596
               ******::* :*::* .. : :*    .  *:    .**:** ::::: :* * *:  .
```

Supplementary Figure S1 (continued)

Parrish et al. (2022), Metabolites

```
P.e.PAL      SYALMLQLREVLVEQALKTPQDEDC-----KEVTPLFTTIPKFEAELKKLLDAEVPKARD   629
E.a.PAL      DYALMLQLRQILVEQTFKVPADSEDESGANSQMPVLFNAIPVFEQALKEALDKEIPKARE   660
P.a.PAL      GYPLMQRLRQVLVEHAIKNPSNERD--EAT----SVMTRIPLFEDELHSQLPAELVNVRA   601
P.n.PAL      ASILMQKLRQVLVDQAMKNVEKEKEKLGAA----STLNRILLFEEELKNLFDSEIPRARE   652
                ** :**::**::::*   ..             :. *  **  *:. :  *: ..*

P.e.PAL      RYDGGDYAVPNRIKNCRTYPIYKFVRGELGTELLRGTATKSPGEDIEKVFTAILDGKLLL   689
E.a.PAL      SYDSGDFAVPNRINNCRTYPLYKFVRSELGTNLLRGTAPRSPGEDIEKVFNGIMEGKLAI   720
P.a.PAL      SFDKGCPPIASKVEECRSFPLYQFVRAQLGTQLLAGTRNQSPGQDFEVVFDAISDGLLMC   661
P.n.PAL      RFDRGQFAVLNRIQNCRTYPLYRFVRDDLGTQLLSGTQTHSPGQDFQKVLDAISEGKLVA   712
              :* *   : .::::**::*:*:*** :***:** **  :***:*:: *: .* :* *

P.e.PAL      PLLKCLEGWRGSAGPFTPRTVP---ASPAAYNPAYWAWFDNVRSPSAT-AGKGYWSLTG-   744
E.a.PAL      PLLRCLEGWRGSAGPFTPRPVP---ASPAAFNPAYWAWFDNIRSPAAM-AGKGSWSSSAF   776
P.a.PAL      PLLQCLEGWTQSPITTC-------------------------------------------   678
P.n.PAL      PLLKCIEGWSGHPGPFSCTQQQQESADIEMCNPSSWIWFDQLHCFKAQGGGKGYFMLSVM   772
             ***:*:***

P.e.PAL      --    744
E.a.PAL      GM    778
P.a.PAL      --    678
P.n.PAL      --    772
```
